# High-Throughput Carbon Substrate Profiling of *Mycobacterium ulcerans* Suggests Potential Environmental Reservoirs

**DOI:** 10.1371/journal.pntd.0005303

**Published:** 2017-01-17

**Authors:** Dezemon Zingue, Amar Bouam, Muriel Militello, Michel Drancourt

**Affiliations:** Aix Marseille Univ, INSERM, CNRS, IRD, URMITE, Marseille, France; University of Tennessee, UNITED STATES

## Abstract

**Background:**

*Mycobacterium ulcerans* is a close derivative of *Mycobacterium marinum* and the agent of Buruli ulcer in some tropical countries. Epidemiological and environmental studies pointed towards stagnant water ecosystems as potential sources of *M*. *ulcerans*, yet the ultimate reservoirs remain elusive. We hypothesized that carbon substrate determination may help elucidating the spectrum of potential reservoirs.

**Methodology/Principal findings:**

In a first step, high-throughput phenotype microarray Biolog was used to profile carbon substrates in one *M*. *marinum* and five *M*. *ulcerans* strains. A total of 131/190 (69%) carbon substrates were metabolized by at least one *M*. *ulcerans* strain, including 28/190 (15%) carbon substrates metabolized by all five *M*. *ulcerans* strains of which 21 substrates were also metabolized by *M*. *marinum*. In a second step, 131 carbon substrates were investigated, through a bibliographical search, for their known environmental sources including plants, fruits and vegetables, bacteria, algae, fungi, nematodes, mollusks, mammals, insects and the inanimate environment. This analysis yielded significant association of *M*. *ulcerans* with bacteria (p = 0.000), fungi (p = 0.001), algae (p = 0.003) and mollusks (p = 0.007). In a third step, the Medline database was cross-searched for bacteria, fungi, mollusks and algae as potential sources of carbon substrates metabolized by all tested *M*. *ulcerans*; it indicated that 57% of *M*. *ulcerans* substrates were associated with bacteria, 18% with alga, 11% with mollusks and 7% with fungi.

**Conclusions:**

This first report of high-throughput carbon substrate utilization by *M*. *ulcerans* would help designing media to isolate and grow this pathogen. Furthermore, the presented data suggest that potential *M*. *ulcerans* environmental reservoirs might be related to micro-habitats where bacteria, fungi, algae and mollusks are abundant. This should be followed by targeted investigations in Buruli ulcer endemic regions.

## Author Summary

Buruli ulcer is a neglected tropical disease which has been reported in over 33 countries, mainly located in tropical and subtropical regions. It is caused by *Mycobacterium ulcerans*, an environmental pathogen associated to slow-moving water. The sources and reservoirs of *M*. *ulcerans* remain elusive and are still to be discovered. In a first attempt to address this issue we used high-throughput carbon substrate profiling of *M*. *ulcerans*. The reported results show that some nutrients, naturally available in organisms present in *M*. *ulcerans’* environment, are metabolized by this microorganism. This carbon substrate determination should help improve the culture of *M*. *ulcerans* as well as suggest potential environmental reservoirs in Buruli ulcer endemic regions.

## Introduction

*Mycobacterium ulcerans* is the etiologic agent of Buruli ulcer, a disabling infection of the cutaneous and subcutaneous tissues [1–3]. *M*. *ulcerans* has been discovered in Bairnsdale, Australia, where Buruli ulcer was initially described [4,5]. Buruli ulcer is a World Health Organization notifiable infection and has been reported at least once by 33 countries located in the rural tropical regions of Africa and South America, in addition to Australia and Japan [6,7]. Over the past ten years, 83.6% (80.89–86.30) of cases were declared by eight West African countries [8]. In these highly endemic regions, the exact reservoirs of *M*. *ulcerans* remain elusive [6, 9–11]. However, epidemiological studies conducted in West African countries all indicated a significant association between the prevalence of Buruli ulcer and the contact of populations with stagnant water sources [12–17] through routine activities such as washing, swimming, fishing and farming [18,19]. A significant progress was recently made by narrowing the possible sources down to contacts with rice fields in Côte d’Ivoire which are sources of stagnant water [16,18,20,21]. Parallel environmental investigations of stagnant water [20,22], water insects [23–25], fishes [26,27] and aquatic mammals [12] showed the presence of PCR-amplified *M*. *ulcerans* insertion sequences (IS) IS*2404*, IS*2606* and KR-B gene. Furthermore, *M*. *ulcerans* partial DNA coding sequences were also recovered from the soil in the vicinity of stagnant water [20,22,26,28,29]. This finding was strengthened by an experimental study confirming a four-month survival of *M*. *ulcerans* in soil [30]. *M*. *ulcerans* DNA has been also detected in water plants [28,31] and in *Thryonhuomys swinderianus* (agouti), a small mammal causing damages to rice fields and in close contacts with rural populations in West Africa [20].

Moreover, this compelling amount of information concerning the presence of *M*. *ulcerans* DNA-related sequences found in the environment has been strengthened by the isolation of five wild strains from those sources [3,32,33].

Here, we propose that a characterization of the metabolic profile of *M*. *ulcerans* may give clues to better define its natural environment including its environmental reservoirs. In this perspective, we used the Biolog Phenotype MicroArray (Biolog Inc., Hayward, CA) for high-throughput carbon substrate profiling of *M*. *ulcerans*. Indeed, Biolog Phenotype MicroArray was previously used to classify and characterize heterotrophic microbial communities from different natural habitats according to their sole-carbon-source utilization profiles [34]. Accordingly, this approach previously unraveled the phenotypic patterns of some *Mycobacterium tuberculosis* complex mycobacteria [35] and *Mycobacterium avium* subsp. *paratuberculosis* [36]. It is used here in the context of unique carbon metabolisms such as chitinase exhibited by *M*. *ulcerans* [37].

## Materials and Methods

### *M*. *ulcerans* strains

This experimental study investigated *M*. *ulcerans* strain CU001 (a gift from Pr V. Jarlier, Paris, France), a clinical isolate representative of the West African epidemic, *M*. *ulcerans* ATCC 19423 isolated in Australia, *M*. *ulcerans* ATCC 33728 isolated in Japan, *M*. *ulcerans* ATCC 25900 isolated in the USA and *Mycobacterium buruli* ATCC 25894 isolated in Uganda [38]. These strains were manipulated into a BLS3 laboratory and a clinical isolate of *Mycobacterium marinum* was isolated in our laboratory [39]. All strains were cultured at 30°C in Middlebrook 7H10 agar medium supplemented with 10% (v/v) oleic acid/albumin/dextrose/catalase (OADC) (Becton Dickinson, Sparks, MD, USA) and 0.5% (v/v) glycerol in a microaerophilic atmosphere for one week for *M*. *marinum* and four weeks for *M*. *ulcerans*.

### Biolog Phenotype microarray

The Biolog Phenotype MicroArray (Biolog Inc.), which consists of 96-well microtiter plates containing each a defined medium that incorporates a unique carbon source (plates PM1 and PM2A for 190 different carbon sources) plus a dye indicator of cell respiration was used, according to the previously reported standard Biolog Inc. protocol [40,41]. *M*. *ulcerans* and *M*. *marinum* colonies were removed from Middlebrook 7H10 medium using a cotton swab previously dipped in 0.1% Tween 80 (WGK Germany, Sigma Aldrich). Mycobacteria were taken with the wet swab off the agar plate culture by gently sweeping on the surface of the culture and then rubbed against the wall of a dry glass tube containing glass beads. The cells were then suspended in GN/GP-IF-0a (Biolog inoculating fluid n°133), the suspension was vigorously vortexed, passed three times through a 29-gauge needle in order to separate aggregates and adjusted to 81% transmittance using a turbidimeter (Biolog Inc). The PM-additive solutions for each plate were prepared according to Table 1. The inoculating fluid (Table 2) consisted of 20 mL of IF-0a GN/GP (1.2 x), 0.24 mL of dye mix G (100x) and 2.0 mL of PM additive (12x) added to the *M*. *ulcerans* or *M*. *marinum* suspension in IF-0a GN/GP (1.76 mL). Each PM plate was then inoculated in duplicate with 100 μL of inoculating fluid. The PM plates were incubated in the OmniLog PM System (Biolog Inc.) which measures the growth of mycobacteria every fifteen minutes for eight days at 30°C. In each well the substrate was reduced to a purple color which was directly proportional to the growth of the mycobacteria. The intensity of the purple color was recorded as dye reduction value, which was then plotted as area under the curve (AUC) by Biolog's parametric software. Negative control wells containing non-inoculated additive solutions in each PM1 and PM2 plates were run at the same time as a quality control element. The threshold separating the wells which exhibited a positive reaction from those with a negative reaction was set for each plate according to the value of the area under the curve (AUC) of the negative control Well (NCW). We defined moderately positive growing wells (MPW) and highly positive growing wells (HPW) as follows: MPW is when the AUC value of the well is equal to or lower than 1.25 times the AUC value of the negative control well, and HPW is when the AUC value of the well is equal to or higher than 1.50 times the AUC value of the negative control. PM plates were further examined visually at the end of each incubation period to ensure an independent verification of the results.

**Table 1:** Composition and preparation of 12 x PM additive solutions.

Ingredient	Final Conc.	120x Conc.	Formula Weight	Grams/ 100 ml	PM 1	PM 2
MgCl_2_, 6H_2_O Ca Cl_2_, 2 H_2_O	2mM 1mM	240mM 120mM	203.3 147.0	4.88 1.76	10 mL	10 mL
Tween 80	0.01%	1.2%	-	1.2	10 mL	10 mL
D-glucose	5mM	600mM	180.2	10.8	-	-
Sterile water					80 mL	80 mL
Total					100 mL	100 mL

**Table 2:** Recipe for 1x PM inoculating fluids from stock solutions.

PM Stock Solution	PM1	PM2
IF-0a GN/GP (1.2x)	20 mL	20
PM additive (12x)	2 mL	2
Dye mix G (100x)	0.24 mL	0.24
cells (13.64x)	1.76 mL	1.76
Total	24 mL	24

### Environmental sources of substrates metabolized by all tested *M*. *ulcerans* strains

In order to find the potential environmental origin of the carbon substrates metabolized by *M*. *ulcerans*, we used the PubMed database to obtain information on the environmental sources for each of the 190 carbon substrates present in the PM1and PM2 plates. The environmental sources were organized in 10 categories (plants, fruits and vegetables, bacteria, algae, fungi, nematodes, mollusks, mammals, insects and the inanimate environment). The Chi-square test was used to compare the proportion of each category for substrates not metabolized by *M*. *ulcerans* versus substrates metabolized by all tested *M*. *ulcerans* strains; a *P* value < 0.05 was used as the criterion for statistical significance. We then used the PubMed database to match each substrate, used as a key-word, with all environmental sources significantly associated with substrates metabolized by all tested *M*. *ulcerans* strains, used as the second key-word (e.g., D-glucosamine and fungi). We calculated the number of hits obtained in this research and compared it to the number of hits obtained by searching only for the key word corresponding to the environmental sources (e.g., fungi).

## Results

### Carbone substrate profiling in *M*. *marinum* and *M*. *ulcerans*

The negative control wells remained negative in all the PMs plates, and results obtained with the five *M*. *ulcerans* strains and the *M*. *marinum* strain were duplicated. A total of 131/190 (69%) carbon substrates were metabolized by at least one of the five *M*. *ulcerans* strains, including 28/190 (15%) carbon substrates common to the five *M*. *ulcerans* strains and 16/190 (8%) carbon substrates metabolized by only one *M*. *ulcerans* strain (Table 3). A total of 21/28 (75%) substrates metabolized by all tested *M*. *ulcerans* strains were also metabolized by *M*. *marinum* (Table 3). In detail, 17/95 (18%) carbon sources in PM1 plates were metabolized by all *M*. *ulcerans* strains and comprised D-glucose-6-phosphate, D-ribose, L-asparagine, uridine, D-fructose-6-phosphate, adenosine, inosine, acetoacetic acid, methyl pyruvate, L-malic acid, D-psicose, L-lyxose, glucuronamide, pyruvic acid, L-galactonic acid-g-lactone, D-galacturonic acid and phenylethylamine. Six of these substrates exhibited a strong positive reaction (D-ribose, L-malic acid, L-lyxose, glucuronamide, pyruvic acid and D-galacturonic acid). Then, 11/95 (11.5%) carbon sources in PM2 plates metabolized by all *M*. *ulcerans* strains comprised D-raffinose, butyric acid, D-glucosamine, α-keto-valeric acid, 5-keto-D-gluconic acid, oxalomalic acid, sorbic acid, L-isoleucine, L-lysine, putrescine and dihydroxyacetone. Five of these substrates exhibited a strong positive reaction (D-glucosamine, 5-keto-D-gluconic acid, oxalomalic acid, sorbic acid and dihydroxyacetone). A total of 21/28 carbon substrates were also metabolized by *M*. *marinum* leaving D-galacturonic acid, uridine, methyl pyruvate, α-keto-valeric acid, L-isoleucine, L-lysine and putrescine as the only substrates specific to *M*. *ulcerans* (Table 3).

**Table 3:** Carbone substrates metabolized by at least one of the five tested *M*. *ulcerans* strains compared with carbon substrates metabolized by *Mycobacterium marinum* on Biolog PM1 & PM2 plates.

Substrates	CU001	ATCC 25900	ATCC 33728	ATCC 19423	ATCC 25894	*M*. *marinum*
D-Ribose						
L-Malicacid						
L-Lyxose						
Glucuronamide						
Pyruvic acid						
D-Galacturonicacid						X
D-Glucosamine						
5-Keto-D-Gluconic acid						
Oxalomalic acid						
Sorbic acid						
Dihydroxyacetone						
Inosine						
L-Galactonic acid-g-Lactone						
D-Raffinose						
Butyric acid						
Putrescine						X
Phenylethylamine						
D-Glucose-6-Phosphate						
Adenosine						
L-Asparagine						
D-Fructose-6-Phosphate						
Acetoacetic acid						
D-Psicose						
α-Keto-Valeric acid						X
L-Isoleucine						X
L-Lysine						X
Methylpyruvate						X
Uridine						X
Fumaricacid						
Tricarballylicacid						
L-Serine						
L-Threonine						
L-Alanine						
L-Alanine-Glycine						
N-Acetyl-β-D-Mannosamine						
Glycyl-L-Proline						
2-Aminoethanol						
3-Methylglucose						
β-Methyl-D-Xyloside						
N-Acetyl-D-Glucosaminitol						
Citramalicacid						
Malonicacid						
Succinamicacid						
3-Hydroxy-2-butanone						
D-Tartaricacid						
L-Tartaricacid						
Acetamide						
L-Arginine						
Glycine						
L-Histidine						
L-Homoserine						
Hydroxy-L-Proline						
L-Leucine						
L-Methionine						
L-Ornithine						
L-Phenylalanine						
L-Pyroglutamicacid						
L-Valine						
D,L-Carnitine						
sec-Butylamine						
D,L-Octopamine						
2,3-Butanediol						
2,3-Butanedione						
Itaconicacid						
D-Lactic acid Methyl Ester						
Melibionicacid						
Oxalicacid						
Quinicacid						
D-Ribono-1,4-Lactone						
Sebacicacid						
Salicin						
Sedoheptulosan						
L-Sorbose						
Stachyose						
D-Tagatose						
Turanose						
Xylitol						
γ-Amino-N-Butyric acid						
δ-Amino Valeric acid						
Capricacid						
Caproicacid						
4-Hydroxybenzoic acid						
β-Hydroxybutyricacid						
γ-Hydroxybutyricacid						
Pectin						
N-Acetyl-D-Galactosamine						
N-Acetyl-Neuraminicacid						
β-D-Allose						
D-Arabinose						
2-Deoxy-D-Ribose						
3-O-β-D-Galactopyranosyl-D-Arabinose						
Gentiobiose						
L-Glucose						
D-Lactitol						
D-Melezitose						
Maltitol						
α-Methyl-D-Glucoside						
2-Deoxyadenosine						
Glycyl-L-Aspartic acid						
Citricacid						
Bromosuccinicacid						
Propionicacid						
Mucicacid						
Glycolicacid						
Glyoxylicacid						
D-Cellobiose						
Glycyl-L-Glutamic acid						
Mono-Methylsuccinate						
D-Malicacid						
Tyramine						
D-Asparticacid						
1,2-Propanediol						
Tween 40						
α-Ketoglutaricacid						
α-Ketobutyricacid						
L-Glutamine						
Tween 80						
α-Hydroxybutyric acid						
β-Methyl-D-Glucoside						
Adonitol						
Maltotriose						
Dulcitol						
D-Serine						
D-Galactonic acid-γ-Lactone						
DL-Malicacid						
Tween 20						
L-Rhamnose						
D-Fructose						
Aceticacid						
α-D-Glucose						
Thymidine						

	Carbone substrates metabolized by at least one of the five tested *M*. *ulcerans* strains.					
	carbon substrates metabolized by only one of the five tested *M*. *ulcerans* strains.					
	carbon substrates metabolized by all tested*M*. *ulcerans* strains.					
	Moderately positive wells					
	Highly positive wells					
X	Carbon substrates which are not metabolized by *M*. *marinum* and metabolized by all tested *M*. *ulcerans* strains.					

### Environmental sources for substrates metabolized by all tested *M*. *ulcerans* strains

Comparing the potential environmental sources in search of substrates metabolized by all tested *M*. *ulcerans* strains versus non-metabolized substrates, we found a significant association between *M*. *ulcerans* metabolized substrates and bacteria (p = 0.000), fungi (p = 0.001), algae (p = 0.003) and mollusks (p = 0.007). The differences were not significant for plants (p = 0.535), fruits and vegetables (p = 0.870), mammals (p = 0.064), insects (p = 0.234) and the inanimate environment (p = 0.477). No carbon source was found to be associated with nematodes. Further MedLine research incorporating bacteria, fungi, algae and mollusks as keywords disclosed that 16/28 (57%) metabolized substrates were associated with bacteria, 5/28 (18%) were associated with alga, 3/28 (11%) were associated with mollusks and 2/28 with fungi. Discarding bacteria because of a potential bias since Biolog was designed for the study of bacterial metabolism, 15/28 (54%) metabolized substrates were associated with fungi whereas 6/28 (21%) were associated with the algae and 6/28 (21%) with mollusks (Table 4).

**Table 4:** Cross-search of the Medline database (May, 2016) for fungi, mollusks and algae as potential sources of carbon substrates; and substrates metabolized by all tested *M*. *ulcerans* strains. The total number of hits for fungi, mollusks and algae is indicated into brackets. Each cell contains the number of cross-hits and green cells indicate the higher relative hit for each carbon source.

	Algae (19292)	fungi (1392904)	Molluscs (52885)	Bacteria (1934745)
D-ribose	24/19292	2571/1392904	114/52885	5133/1934745
Glucuronamide	0/19292	4/1392904	0/52885	7/1934745
D-Galacturonicacid	0/19292	79/1392904	3/52885	218/1934745
D-Glucosamine	8/19292	498//1392904	28/52885	4499/1934745
Oxalomalic acid	0/19292	0/1392904	0/52885	0/1934745
Sorbic acid	0/19292	357/1392904	2/52885	411/1934745
Dihydroxyacetone	7/19292	294/1392904	3/52885	504/1934745
L-Galactonic acid-g-Lactone	0/19292	0/1392904	0/52885	0/1934745
D-Raffinose	3/19292	646/1392904	4/52885	728/1934745
Butyric acid	26/19292	3089/1392904	266/52885	5689/1934745
Putrescine	17/19292	1174/1392904	27/52885	2226/1934745
Phenylethylamine	10/19292	683/1392904	225/52885	908/1934745
D-Psicose	1/19292	14/1392904	0/52885	77/1934745
L-Malicacid	1/19292	107/1392904	0/52885	301/1934745
L-Lyxose	0/19292	4/1392904	0/52885	28/1934745
Pyruvic acid	19/19292	686/1392904	19/52885	1484/1934745
5-Keto-D-Gluconic acid	0/19292	5/1392904	0/52885	21/1934745
Inosine	2/19292	999/1392904	37/52885	1540/1934745
D-Glucose-6-Phosphate	1/19292	57/1392904	2/52885	94/1934745
Adenosine	182/19292	14109/1392904	1029/52885	27257/1934745
L-Asparagine	17/19292	1980/1392904	37/52885	3428/1934745
D-Fructose-6-Phosphate	2/19292	7/1392904	0/52885	92/1934745
Acetoacetic acid	0/19292	20/1392904	1/52885	62/1934745
a-Keto-Valeric acid	0/19292	2/1392904	0/52885	10/1934745
L-Isoleucine	4/19292	1400/1392904	40/52885	3296/1934745
L-Lysine	65/19292	6989/1392904	254/52885	11894/1934745
Methyl pyruvate	7/19292	4/1392904	0/52885	7/1934745
Uridine	30/19292	3408/1392904	112/52885	6435/1934745

## Discussion

We determined that five different strains of *M*. *ulcerans* could use 28 different substrates as sources of carbon. These results were authenticated by the negativity of the negative controls introduced in every plate and the reproduction of data over two replicates. Moreover, stringent criteria were used to ensure the predictive value of the positive results. However, only seven of these 28 substrates were found to be specifically used by *M*. *ulcerans* and not by the phylogenetically closest species *M*. *marinum*. Three of these seven carbon sources indeed contain indispensable amino-acids.

The carbon sources here determined for *M*. *ulcerans* may by incorporated in culture media in the perspective of enhancing the isolation and culture of this pathogen. Indeed, *M*. *ulcerans* is a slow-growing mycobacterium and the availability of an improved method for its culture would improve the diagnosis of Buruli ulcer patients and the quest for environmental reservoirs [32]. As an example, it has been shown that the incorporation of chitin into the Middlebrook 7H9 broth enhances the growth of *M*. *ulcerans* [37]. Accordingly, our study points towards a possible association of *M*. *ulcerans* with fungi as a potential source of chitin, a polysaccharide possibly degraded by *M*. *ulcerans’* genome-encoded chitinase [42]. Likewise, the other carbon sources here disclosed should be tested for their potential to increase the cultivation of *M*. *ulcerans*.

Moreover, our analyses suggested that *M*. *ulcerans* may have found some sources of carbon in microbial communities including alive and dead bacteria, fungi and algae. As for bacteria, it has been previously reported that *M*. *ulcerans* was isolated in environments where 17 other mycobacteria species were also isolated, including *M*. *fortuitum* as a constant co-inhabitant [3, 32, 33]. These results suggest cross-feeding between various bacterial complexes including mycobacteria, for the acquisition of carbon. Likewise, green algae extracts have been shown to halve the *in vitro* doubling time of *M*. *ulcerans* and promote the formation of biofilm [31]. We observed that *M*. *ulcerans* metabolizes D-galacturonic acid, the main component of pectin contained in the primary cell walls of terrestrial plants, and putrescine, a foul-smelling chemical derived from the decomposition of dead plants, which indicates that *M*. *ulcerans* may live in assemblages of dead aquatic plants. This finding is reinforced by the observation that *M*. *ulcerans’s* genome encodes five putative cutinases. Cutinases are mainly produced by phytopathogenic fungi to hydrolyze cutin (a main component of the cuticle which covers the aerial surfaces of plants) during plant colonization process [43].

Green algae are among the main food of freshwater mollusks pointed out in our study; mollusks are herbivores like other species of the freshwater snail family [44]. The principal genera of mollusks met in freshwater in West Africa are *Bulinus*, *Planorbis*, *Pila*, *Lanistes*, *Melania*, *Bithynia*, *Lymnaea*, *Biomphalaria*, *Mutela*, *Aspatharia* and *Sphaerium* [23,45]. Previous molecular investigations reported the detection of specific *M*. *ulcerans* DNA sequences in *Bulinus* spp. [23,46], in *Planorbis* spp. [23] and in mollusks of different Gastropoda order, Bivalvia order and Basommatophora order [26]. Furthermore, the experimental infection of *Pomacea canaliculata* (Ampullariidae) and *Planorbis planorbis* (*Planorbidae*) by plants contaminated by *M*. *ulcerans*- showed through optic microscopy digestive tract observation that snails remained infected by viable mycobacteria up to 25 days [23]. Small mollusks are also known to be a prey for water bugs which are involved in the transmission of *M*. *ulcerans* in Buruli ulcer endemic regions [3]. In West Africa, approximately 76% of the population lives next to rivers, lakes, and other water bodies contaminated with intermediate hosts such as snails [47].

In conclusion, our study is suggesting paths to improve culture media for the enhanced isolation of *M*. *ulcerans* by mimicking the natural ecosystem of *M*. *ulcerans* which is probably living in microbial communities with other bacteria, fungi and algae. These data support the recent hypothesis that mollusks could be part of a larger food chain including several hosts giving appropriate shelters to *M*. *ulcerans*, as recently reported [48]. Small mollusks should be further investigated using culture-based appropriate methods in the search for *M*. *ulcerans*.
